# The inhibitory control of pheasants (*Phasianus colchicus*) weakens when previously learned environmental information becomes unpredictable

**DOI:** 10.1007/s10071-019-01328-4

**Published:** 2019-12-16

**Authors:** Kandace R. Griffin, Christine E. Beardsworth, Philippa R. Laker, Jayden O. van Horik, Mark A. Whiteside, Joah R. Madden

**Affiliations:** Centre for Research in Animal Behaviour, Psychology, University of Exeter, Exeter, EX4 4QG UK

**Keywords:** Pheasant, *Phasianus colchicus*, Inhibitory control, Detour reach task, Predictability, Unpredictable environment

## Abstract

Inhibitory control (IC) is the ability to intentionally restrain initial, ineffective responses to a stimulus and instead exhibit an alternative behaviour that is not pre-potent but which effectively attains a reward. Individuals (both humans and non-human animals) differ in their IC, perhaps as a result of the different environmental conditions they have experienced. We experimentally manipulated environmental predictability, specifically how reliable information linking a cue to a reward was, over a very short time period and tested how this affected an individual’s IC. We gave 119 pheasants (*Phasianus colchicus*) the opportunity to learn to associate a visual cue with a food reward in a binary choice task. We then perturbed this association for half the birds, whereas control birds continued to be rewarded when making the correct choice. We immediately measured all birds’ on a detour IC task and again 3 days later. Perturbed birds immediately performed worse than control birds, making more unrewarded pecks at the apparatus than control birds, although this effect was less for individuals that had more accurately learned the initial association. The effect of the perturbation was not seen 3 days later, suggesting that individual IC performance is highly plastic and susceptible to recent changes in environmental predictability. Specifically, individuals may perform poorly in activities requiring IC immediately after information in their environment is perturbed, with the perturbation inducing emotional arousal. Our finding that recent environmental changes can affect IC performance, depending on how well an animal has learned about that environment, means that interpreting individual differences in IC must account for both prior experience and relevant individual learning abilities.

**Electronic supplementary material:**

The online version of this article (10.1007/s10071-019-01328-4) contains supplementary material, which is available to authorized users.

## Introduction

Executive functions are the general processes that control and regulate an individual’s thoughts and actions (Miyake and Friedman 2012). One facet of executive function is inhibitory control (IC), which is the capacity to deliberately resist pre-potent or dominant responses, described as impulsive actions (Frijda 2010), and it makes up an integral part of self-regulation (Friedman et al. 2008; Mischel et al. 2010). Within humans, poor IC has been associated with behavioural problems and psychopathological disorders (Dalley and Robbins 2017; Hamilton et al. 2015). In both humans and non-human species, individuals vary in the level to which they can exert IC (Friedman et al. 2008; Meier et al. 2017; Mittal et al. 2015; Miyake and Friedman 2012; Völter et al. 2018). Individual variation in IC may be considered a stable trait explained by both genetic and developmental factors, leading to performances that are consistent across time. In healthy human adults, this stability may be partly due to hereditary influences (Friedman et al. 2008; Miyake and Friedman 2012). Additionally, adults who had been raised in unpredictable environments during childhood showed reduced IC abilities compared to those who experienced a more stable upbringing (Mittal et al. 2015). Consequently, it might be assumed that IC is variable across individuals but once established, is relatively stable.

An individual’s ability to exert IC may, however, also be plastic and thus susceptible to short-term, recent experiences. Transitory emotional and motivational factors contribute to individual differences in IC (Botvinick and Braver 2015; Pessoa 2009); for example, Padmala and Pessoa (2010) showed in humans that responses which were previously rewarded were harder to suppress than previously unrewarded responses. People can become less cautious and act faster after experiencing bad outcomes (Amsel 1958; Verbruggen et al. 2017). These effects may be ephemeral. Training with stop-signal IC tasks reduced monetary risk taking up to 2 h after the training regime (Verbruggen et al. 2012), but the effect declined after a 24 h gap (Verbruggen et al. 2013). In dogs, IC also appears to depend on an individual’s state at least for breeds that have not been selected for low arousal, with highly aroused pet dogs performing poorly in an IC task (Bray et al. 2015). An individual’s condition may influence their motivational state, thus affecting their behaviour: North Island Robins/Toutowai (*Petroica longipes*) with poor body condition performed poorly on an IC task (Shaw 2017). It seems likely that variation in IC across individuals, partially determined by genetic or developmental factors, may also be explained by fluctuations in their motivational state affected for a short period by recent experience.

Given that IC involves evaluating sets of information and deciding which set to act upon, their understanding of the reliability of information or more generally the predictability of their environment is likely to shape an individuals’ IC performance (Verbruggen et al. 2016). When information is consistent and the environment is predictable then we expect that individuals will exert IC and switch actions to predictably better alternatives. When information is inconsistent and the environment is unpredictable, it is expected that IC will be weakened as individuals discount alternatives and become more impulsive (Frankenhuis et al. 2016). Individuals are expected to vary their levels of IC as the environment changes, and this variation may occur rapidly over short time scales as the value of information fluctuates. It is important to understand the plasticity of an individual’s IC from an evolutionary perspective because IC contributes to navigation, survival and other behaviours with fitness consequences in the natural environment (MacLean et al. 2014). Understanding whether an individual’s IC performance is a stable trait or more temporally variable and state-like will determine how selection pressures can act upon it.

Measuring IC in non-humans has been achieved using a range of different methods. They may include detour-reach tasks (e.g. Boogert et al. 2011; Bray et al. 2015; Vernouillet et al. 2016), A-not-B tasks (e.g. Bray et al. 2014; Jelbert et al. 2016; MacLean et al. 2014; Osthaus et al. 2013), reversal-learning tasks (e.g. Beran et al. 2008; Boogert et al. 2011; Floresco et al. 2008; Shaw et al. 2015) and stop-signal tasks (e.g.Beuk et al. 2014; Lacreuse et al. 2016; Liu et al. 2009; Meier et al. 2017). Extracting a robust measure of an individual’s IC ability may be complicated by their motivation and prior experience (van Horik et al. 2018d). Different tasks may reflect different aspects of IC, with poor correlations between performances in different tasks within individuals, even though each task is ostensibly designed to reveal IC (Brucks et al. 2017; Vernouillet et al. 2018; Völter et al. 2018). Indeed, subtle variants of the test apparatus, such as its size, can alter measures of an individual’s IC (Bobrowicz and Osvath 2018). The most commonly used test of IC deployed across a range of non-human animals is the detour tube task, used in > 40 species that we are aware of and subject to a number of validatory studies [reviewed in (Kabadayi et al. 2018)]. This task involves training an individual to approach an opaque cylinder from the side such that the reward (usually food) cannot be seen. Once the individual learns to acquire the reward from the open ends of the cylinder without touching the sides, they are presented with an identical but transparent cylinder such that the reward is visible as they approach. Thus the reward appears to be accessible through the transparent barrier. Inhibitory control is required to complete the task because the visibility of the reward creates a tendency to reach for it directly, whereas subjects should inhibit this tendency and detour around the barrier (Kabadayi et al. 2018). Levels of IC may be indicated by two (likely correlated) behaviours. The number of erroneous pecks made at the barrier (used by, e.g. Lucon-Xiccato and Bisazza 2017) provides a measure of how much effort an individual will exert on an unrewarded action. The time taken to circumvent the barrier to attain the reward (used by, e.g. Vernouillet et al. 2018) may provide a measure of how long an individual will persist in an unrewarded behaviour but it may also reflect non IC aspects such as the speed or agility of the individual. Poor IC may be revealed by repeated, unrewarded contacts (touches, pecks, etc. depending on the taxa being studied) with the cylinder, indicating attempts to access the reward within. Poor IC may also be revealed by a high latency to access the reward if the individual takes a long time to move away from the unprofitable direct approach to the reward. An individual exhibiting high IC will refrain from the pre-potent pecking behaviour and will detour around the barrier to reach the reward through the open end of the cylinder, exhibiting the previously learned movement pattern.

Pheasants (*Phasianus colchicus*) provide an established non-human animal study species to explore individual variation in the expression of IC and previous research has utilised three different methods of assessing IC. First, on two detour tasks (one barrier and one tube), pheasants with narrower dietary breadth performed better than individuals with a wider dietary breadth (van Horik et al. 2018a). Individual pheasants improved their IC performance with prior experience of transparent barriers, demonstrating the importance of controlling for previous experience when utilising detour reach tasks (van Horik et al. 2018a). Individual variation in IC performance on this task may be shaped by their early life physical environment that the birds grew up in, with birds that grew up in a spatially unpredictable environment in which barriers were randomly moved each day exhibiting better IC (fewer unrewarded pecks at the transparent barrier) (van Horik et al. 2019). Second, during a colour acquisition and reversal task, pheasants initially had difficulty resisting the previously learned association during the reversal phase, but their performance improved over time. Therefore, individuals did learn to inhibit their initial response to the previously learned association rule (van Horik et al. 2018b), and individuals that were slow to reverse this learned association survived better after release into the wild (Madden et al. 2018). However, this task is susceptible to initial cue biases and because it takes multiple trials necessary for the initial learning of the association and its subsequent reversal, it is also susceptible to unexpected disturbances to testing conditions that affect the attention or motivation of test subjects in one or more trials. Third, on a stop-change motor task, IC performance was influenced by morphology and sex (Meier et al. 2017). Smaller pheasants had better IC scores than larger individuals. In addition, males performed better than females; specifically, males displayed more movement regulation on the task, turning earlier towards the new reward site. These differences may result from IC ability or the physical constraints of the task (Meier et al. 2017). Pheasants might be expected to require IC in a wide variety of contexts such as response to predators, mate choice, movement in the natural landscape and appropriate food selection. In addition to prior work measuring IC in the species, pheasants offer the logistical advantage of being precocial, such that they can be artificially reared in large numbers in the absence of adults (ensuring homogeneity of controlled rearing environments and removing non-genetic parental influences), their diet can be standardised and chicks grow rapidly so that they can participate in behavioural tests within a few weeks of hatching. These features permit researchers to have complete control over their early life experiences and standardise tasks across age-matched cohorts.

We gave pheasants the opportunity to learn an associative rule in a binary choice task, and simultaneously trained them on a detour reach task. We assessed the extent to which each individual had learnt the rule by calculating their probability of making a correct choice on their final, 80th, choice. We then perturbed this rule for half of the birds, creating a temporarily unpredictable environment, while the other half of the birds continued to experience the normal rule. Individuals were immediately presented with a detour reach task and their IC responses measured, which allowed us to test whether our perturbation affected either: their time to interact with the test apparatus (measured as the time to take a freely accessible reward on the apparatus); their time to take the reward worm; and the number of erroneous pecks that they made to the transparent apparatus. Specifically, we tested whether their performance in this detour task was affected by the extent to which they had learnt the task and the amount of experience that they had with the tube apparatus in conjunction with their perturbation history. Three days later all birds were presented with the discrimination task with the original affordances and were again presented with the detour reach task and their IC responses measured to test whether the single instance of perturbation had persistent effects.

## Methods

One-hundred and twenty-eight pheasant chicks were hatched on the same day and housed at Rothamsted Research North Wyke, in four replicated enclosures, in groups of 32 between 24 May 2018 and 25 July 2018. The chicks had been hatched from eggs collected from groups of breeding adults that contained some individuals that we had bred in the previous year, released into the wild and then recaptured a year later, as well as other adults that we had not bred and thus were either wild-born or immigrants from shooting estates nearby. All individuals were fed age-appropriate commercial pheasant feed (Keeper’s Choice) and provided water ad libitum. Birds were individually identified through numbered wing tags. For the first 2 weeks of life, each group of chicks was housed in heated enclosures (2 × 2 m). The following week, chicks were given additional access to covered, unheated outdoor enclosures (1 × 4 m). For the final 7 weeks of rearing, individuals had further access to 4 × 12 m outdoor enclosures.

### Procedures

#### Experimental overview

**Fig. 1:**
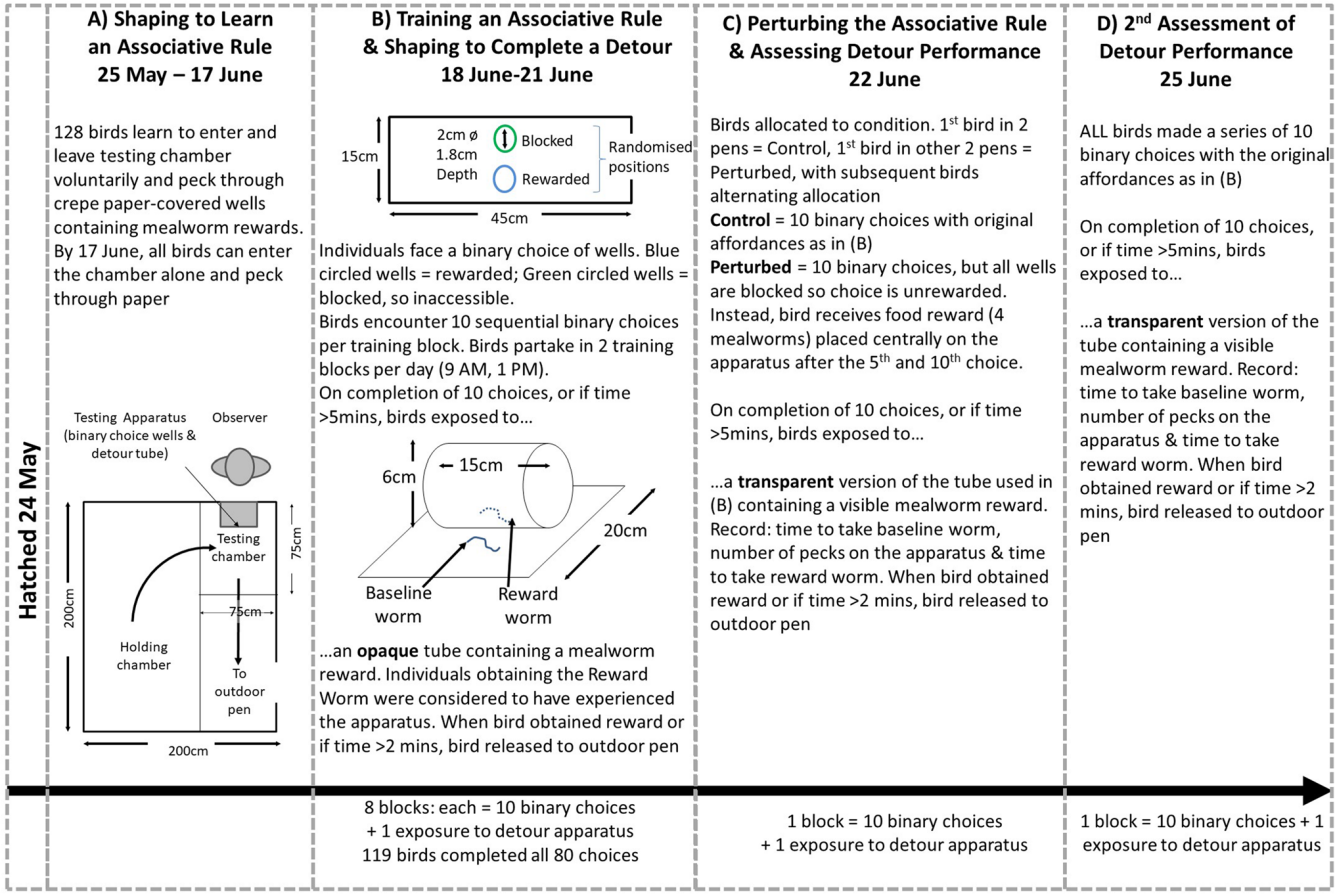
A timeline of the experimental procedure including illustrations of the housing and testing apparatus

Chicks were shaped from 1-day-old to voluntarily enter a testing chamber and operate apparatus which would be used to train them to learn an associative rule (Fig. 1a). When 4 weeks old, the pheasants had the opportunity to learn an associative rule in which, during a binary choice task, one visual cue was always rewarded and a second cue was always unrewarded (Fig. 1b). Individuals experienced 80 choices arranged in eight blocks of 10 choices over 4 days. During this time, they were shaped to access a reward from an opaque cylinder which was presented to them immediately after they had completed each block. We then randomly allocated pheasants to one of two conditions so that in a ninth block, half of the individuals received exactly the same task as during their training period (unperturbed control) while the other half received a task that appeared identical (the same cues were presented in the same manner) but in which the original rule was disrupted as neither well was rewarded (perturbed condition) (Fig. 1c). Immediately after the ninth block, both sets of birds (control and perturbed) were presented with a transparent version of the cylinder used during the training blocks in which the reward was visible but accessible only by their making a detour to either end to reach inside. Their time to take a freely available ‘baseline’ worm provided a measure of their motivation to interact with the apparatus. The number of erroneous pecks made at the transparent barrier, and the time taken to circumvent the barrier to attain the reward, are considered to provide measures of IC (see above). Three days later, we presented a final 10th block of choices to all birds, with the apparatus having the affordances of the initial training blocks with one cue being rewarded and one cue being unrewarded (Fig. 1d). Immediately after making these choices, we once again presented a transparent version of the tube and recorded their interactions with the apparatus. This allowed us to test if the effect of the initial perturbation persisted over several days.

#### Shaping to learn an associative rule (Fig. 1a)

From first day post birth, pheasants were habituated to experimenters and shaped to voluntarily enter a visually isolated testing arena (0.75 × 0.75 m) that was separated from the holding chamber by a sliding door, where they were shaped to obtain a concealed food reward (single mealworm) from a test apparatus comprising a series of wells (diameter 2 cm, depth 1.8 cm) covered in crepe paper. The apparatus (45 × 15 cm) contained ten wells on a white platform. Individuals were shaped in groups over their first 2 weeks to peck through the crepe paper. This was achieved progressively by initially presenting mealworm rewards in open wells without colour cues, then presenting a mix of open and partially covered wells, and then a mix of partially and fully covered wells and finally fully covered wells. Once all the wells had been opened, birds were allowed to voluntarily exit to the outdoor pen. All individuals were assessed in isolation during their third week and all included in this study were able to open wells and obtain the mealworms within.

#### Training an associative rule (Fig. 1b)

Individuals were presented with a binary choice colour discrimination task. The apparatus was modified to expose just two wells, placed centrally, one above the other. Each well was surrounded by a 5 mm-wide band of colour and sealed with crepe paper. The wells encircled by blue contained a mealworm reward, while the green encircled wells were made inaccessible by placing a layer of hard black card beneath the crepe paper. If individuals made a correct choice, they were allowed to eat the reward mealworm before the experimenter revealed two new wells. If an incorrect choice was made, both wells were removed and two new wells were presented. The rewarded well’s location (top or bottom) was pseudorandomised across the choices, and it did not occur in the same position for more than three consecutive choices (van Horik et al. 2018b). For each pair of wells, we recorded whether the bird made a correct or incorrect choice. Choices were presented in blocks of 10. Each individual participated in 2 blocks per day starting at 9 am or 2 pm. When the individual had completed their 10 choices or if they did not interact with the task within 5 min, they were presented with the detour test apparatus.

We generated learning curves for individuals that had completed all 80 choices (Langley et al. 2018) and from these we calculated their predicted probability of making a correct decision on their first and last choice. We used a binary logistic regression model (GLM) that considered weather a bird made a correct choice in each of their 80 choices or not. From these curves we calculated the probability that an individual would choose correctly on their final trial (*X* = 80), which is derived from solving the equation *Y* = 1/(1 + exp[− (*b*_0_ +* b*_1_*X*)]), where*b*_0_ depicts the intercept and *b*_1_ depicts the slope estimate from their learning curve GLM. We consider this measure indicative of the extent to which an individual has learned the task by the end of the testing. Individuals that have a probability of > 50% on their final discrimination might be considered to have learnt something, at least partially, about the task. However, one could also apply a more conservative criteria in which only individuals that exceed a certain threshold probability are deemed to have learnt the task. Commonly, in traditional criteria-based assessments of learning, a threshold of 80% correct choices is used. Therefore, we repeated our analysis including only birds that exhibited a probability of > 80% of making the correct choice on their 80th discrimination.

#### Shaping to complete a detour (Fig. 1b)

Individuals were shaped to complete a cylinder detour task. This established that the individuals possessed the necessary motor skills needed to reach inside the cylinder and retrieve the reward. On completion of their training on the associative rule (see above), the observer placed the training cylinder on top of the well apparatus. The training cylinder (15 cm long, 6 cm diameter) was open on both ends, which required individuals to walk to the side of the cylinder to obtain the fresh killed mealworm reward placed inside the apparatus at the centre point of the cylinder. The cylinder was mounted centrally on a white Perspex square (20 × 20 cm) and a fresh-killed mealworm was placed in front of the apparatus (baseline worm) to centre the focal individual to the apparatus at the start of each trial (van Horik et al. 2018a). When the bird obtained the reward worm it was allowed to voluntarily exit to the outdoor pen and was considered to have gained one instance of experience of the apparatus. If the bird did not gain the reward worm within 2 min, it was allowed to voluntarily exit to the outdoor pen and was considered to have not gained an instance of experience of the apparatus.

#### Perturbing the associative rule (Fig. 1c)

On 22nd June, after the last of the eight sessions that trained the associative rule, we conducted the experimental manipulation. As usual, birds entered the test chamber of their own volition and we alternated whether an individual was faced with a ‘normal’ associative task or one in which the rule was perturbed to control for any testing order effects. Half the birds in each pen experienced a perturbed task while the other half received an unperturbed control task. The Control condition matched exactly a single block of 10 choices with the cue affordances being identical to those used during training. The Perturbed condition involved the birds being faced with an identical test apparatus to that used during training comprising one block of 10 choices, but in this condition all of the wells were blocked so that they could be pecked at but no mealworm reward was accessible. This meant that the affordances of the cues that could be learned during training were lost. To control for potential effects of hunger and motivation, individuals in the Perturbed condition were provided with four freely attainable mealworms, placed on the apparatus between the two wells after each of choice 5 and choice 10 of the colour discrimination presentations. This matched the quantity of food reward they would have obtained had they made 80% correct choices in the normal (control) task. The first bird to enter the testing chamber in two of the four pens received the Control condition while the first bird entering in the other two pens received the Perturbed condition. Subsequent birds were given alternating conditions. When the individual had completed their ten choices they were presented with the detour test apparatus.

We chose to use a perturbation in which no choice was rewarded rather than a reversal in which the choice outcomes were switched (which would also have perturbed the information provided by the cues) because we believed that conducting a reversal could have complicated our interpretation of the results. First, if an individual had learned (to any extent) the reversal within their ten choices in their ninth block then they may not have experienced the same level of frustration as those that failed to learn it. Second, a reversal would have complicated our delivery of a standardised food reward to birds. This would have confounded hunger levels and thus may have influenced motivation, making our results hard to interpret. By ensuring that all perturbed birds experienced blocked wells, preventing them from getting any mealworms from the previously rewarding wells, we could ensure that all birds were equally frustrated and ate an equal amount of mealworms immediately prior to the detour task.

#### Assessing detour performance (Fig. 1c)

We presented the same detour apparatus, but this time, the cylinder was transparent, permitting individuals to see the reward through the clear plastic, but requiring them to resist their pre-potent response to peck toward the mealworm through the cylinder. Individuals were required to resist their pre-potent response to peck toward the mealworm through the cylinder, and instead detour to the sides of the cylinder as they had learned with the opaque cylinder. We recorded the time to take the baseline worm as a measure of motivation to interact with the apparatus, the time to take the reward worm after having taken the baseline worm, and the number of pecks at the apparatus. When the bird obtained the reward worm, or if it did not gain the reward within 2 min, it was allowed to voluntarily exit to the outdoor pen.

#### A second assessment of detour performance (Fig. 1d)

On 25th June, following 2 days during which time birds did not experience any further shaping or testing, we presented all birds with the discrimination apparatus used to train the associative rule (see above) with the original affordances such that blue encircled wells were rewarded and green encircled wells were blocked. Each bird was presented with one block of ten choices. When the individual had completed their ten choices or if they did not interact with the task within 5 min, they were presented with the transparent detour test apparatus and we assessed their detour performance as described above.

#### Motivation, morphology and sex

During each session, the order in which individuals entered the testing arena within each enclosure was recorded and we calculated a mean score across the sessions for each individual. This test order (TO) score provides a measure of motivation, with lower scores indicating earlier entry into the testing arena and higher motivation (van Horik et al. 2017). After testing was complete, mass was recorded with a Slater Super Sansom spring balance scale (precision 5 g), their tarsus length measured with callipers (precision = 0.1 mm) and their body condition calculated as mass/tarsus^3^. At the end of the 10-week rearing period, sex was determined using visual cues.

### Statistical analysis

All statistical analysis was performed in R v. 3.5.1 using the lme4 package (Bates et al. 2014). One hundred and nineteen of the birds completed the full 80 choices and so were included in the analysis. We used Generalized Linear Mixed Models (GLMMs) fitted with a Poisson distribution because peck data are counts and our latency measure was capped at 300 s. We ran three separate models with (a) the time to take the baseline worm; (b) the time to take the reward worm; (c) the number of unrewarded pecks made during the detour task as each of the dependent variables. For each model we included the following as main effects: whether the individual experienced a control or perturbed condition [PERTURB], the individual’s predicted probability of making a correct choice on their last choice calculated from the learning curves [LEARNING], the total number of sessions that it interacted with the opaque cylinder prior to testing/10 [EXPERIENCE], the individual’s body condition score *100 [CONDITION], sex [SEX] and whether the behaviour was being measured in the first (22 June) or second (25 June) test period [PERIOD]. We also included 3-way interactions [PERTURB:LEARNING:PERIOD and PERTURB: EXPERIENCE:PERIOD] to test whether overall, the effect of perturbation on behaviour was affected by learning or prior experience differently in the two time periods. We included the 2-way interactions underlying these 3-way interactions. We included individual ID nested within their rearing pen as random factors because we took repeated measures of the same individual. If models initially failed to converge, we increased iterations to 10,000 and used a ‘bobyqa’ optimizer. We visually checked residuals from each model for normality and homoscedasticity. We adjusted two of our variables so that all variables fell within the same order of magnitude to improve model fitting. This involved our dividing the total number of sessions by ten and multiplying an individual’s body condition score by 100. We did not reduce models. Post-hoc tests (Tukey) were conducted using the package lsmeans (Lenth 2016).

### Ethical considerations

All work was performed under Home Office license PPL 30/3204 to JRM. To minimise stress, pheasants were habituated to human observation from 1  day post birth, and we utilised shaping protocols to familiarise individuals with the testing arena and test apparatus. The shaping procedure encouraged individuals to participate on their own volition, and individuals chose whether to participate or not. No aversive stimuli were imposed.

## Results

### Did individuals learn the associative rule?

1. Individuals increased from a mean probability of 0.496 (95% CI 0.473–0.518) of making the correct choice on their first discrimination to a mean probability of 0.805 (95% CI 0.788–0.822) of making the correct choice on their 80th discrimination (Paired *t* test *t*_116_ = 18.6, *p* < 0.001, Fig. 2). The probability of making a correct choice on the 80th discrimination did not differ between individuals destined to be control or perturbed (*t* test *t*_115_ = 1.46, *p* = 0.153). In the subsequent analyses we only included individuals that had a probability of > 50% of making a correct choice in their final discrimination; thus we could describe them as showing some evidence of improvement on the original probability and, therefore, they could be considered to exhibit some degree of learning of the task. This definition of learning is debatable and it may be more informative to consider only those birds that have exceeded a criteria considered to indicate that they have learnt the task. We adopted this more conservative approach and repeated all the subsequent analyses on a subset of 59 birds that exhibited a probability of > 80% of making the correct choice on their 80th discrimination. We report these analyses in the ESM, but they do not differ qualitatively from the effects that we detect when considering individuals exhibiting the broader spectrum of probabilities.

**Fig. 2:**
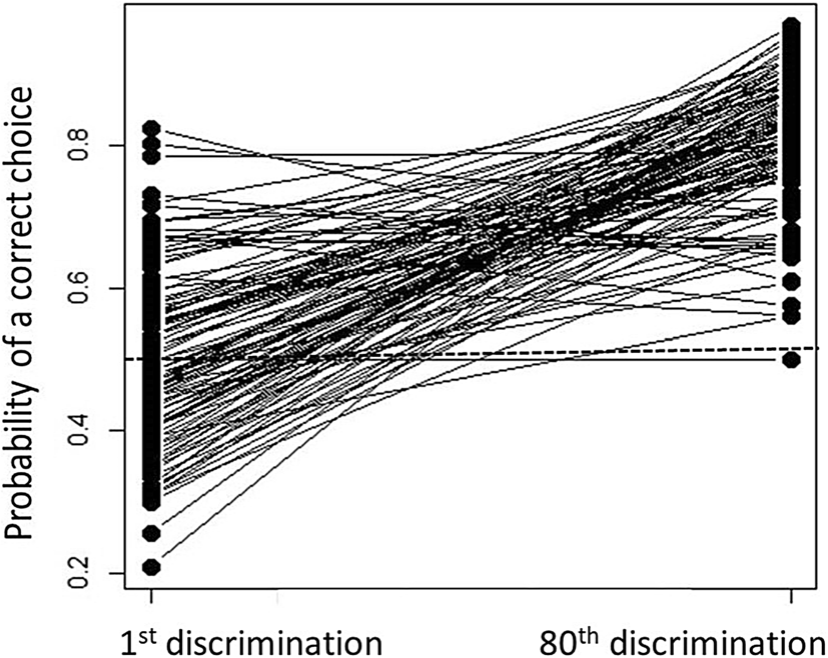
An individuals’ probability of correctly choosing the rewarded well in a binary choice, derived from the slope of their learning curve, on their first and last (eightieth) discrimination. The dashed line indicates chance performance (50%)
2. What influenced an individual’s time to interact with the detour test apparatus?
  Individuals that had engaged more times with the opaque cylinder during the training blocks were faster to take the baseline worm during both the test periods with the clear cylinder (Table 1, Fig. 3a). Individuals that had poorer body condition were faster to take the baseline worm during both test periods (Table 1, Fig. 3b). Perturbation had no effect on an individual’s time to take the baseline worm, either overall or across the two test periods (Table 1). There was no effect of sex or prior learning rate on time to take the baseline worm.

**Table 1:** Model output from a GLMM testing the relationship between the time to take a baseline worm and a suite of individual, temporal and environmental variables

Predictors	Baseline worm		
	Estimate	SE	*p*
(Intercept)	0.58	1.23	0.559
Perturb	− 0.37	1.47	0.715
Learning	0.03	1.27	0.978
Experience	− 3.99	0.38	**< 0.001**
Sex	0.74	0.15	0.457
Condition	2.01	2.51	**0.045**
Period	0.90	1.04	0.369
Perturb:period	− 1.23	1.52	0.220
Perturb:learning	− 0.25	1.76	0.804
Perturb:experience	1.04	0.59	0.299
Perturb(control):learning:period	− 1.10	1.27	0.270
Perturb(perturbed): learning:period	1.07	1.32	0.287
Perturb(control):experience:period	1.52	0.33	0.128
Perturb(perturbed):experience:period	0.44	0.47	0.658
Random effects			
*σ*^2^	0.40		
*τ*_00_ _Individual:Pen_	0.26		
*τ*_00_ _Pen_	0.00		
ICC _Individual:Pen_	0.40		
ICC _Pen_	0.00		
Observations	233		

For factors, perturb is set to perturbed; sex is set to male; period is set to the second period; unless specified in parentheses

Significant values (P < 0.05) are indicated in bold

**Fig. 3:**
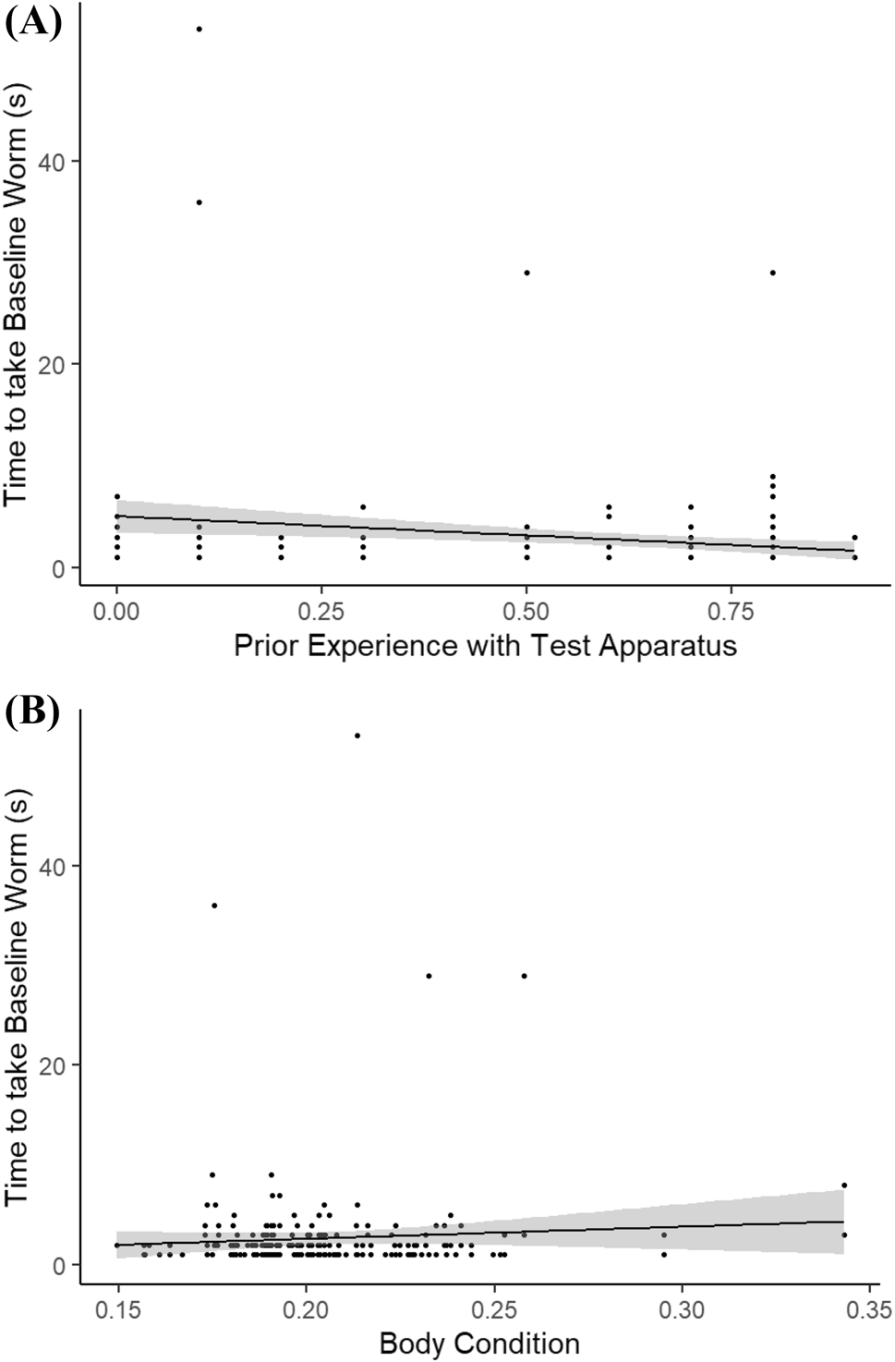
The relationship between the time that it took a focal individual to take and eat the baseline worm placed on the detour test apparatus during their testing periods and: **a** the level of experience that the individual had with the detour apparatus during the preceding shaping blocks (value is the raw experience score/10); **b** their body condition (value is the raw body condition score × 100). Lines indicate linear best fits. Shaded areas indicate 95% CI
3. What influenced the time it took for an individual to access the reward worm?
  During the first test period, perturbed individuals, but not control individuals, that had exhibited poor learning of the discrimination task took longer to access the reward worm, whereas this difference was not seen in the second period (Table 2, Fig. 4a). Individuals that had more experience with the detour test apparatus during shaping were faster to access the reward worm, but this effect was seen to be strongest for control individuals during the second period (Table 2, Fig. 4b). The overall decrease in time to obtain the reward between periods was significant for both perturbed and control individuals (post hoc Tukey tests both *p* < 0.0001) and but overall, both groups took the same time to access the reward during the first period (post hoc Tukey test *p* = 0.118) and second period (post hoc Tukey test *p* = 0.987). There were no effects of sex or condition on time to take the reward worm (Table 2).

**Table 2:** Model output from a GLMM testing the relationship between the time taken (s) to access the reward worm placed inside a transparent cylinder, and a suite of individual, temporal and environmental variables

Predictors	Time to access the reward worm		
	Estimate	SE	*p*
(Intercept)	2.82	1.90	**0.005**
Perturb	1.77	2.11	0.077
Learning	0.98	1.87	0.326
Experience	− 4.90	0.63	**< 0.001**
Sex	− 0.24	0.25	0.810
Condition	− 1.55	4.32	0.121
Period	3.06	0.35	**0.002**
Perturb:period	− 11.31	0.42	**< 0.001**
Perturb:learning	− 1.92	2.48	0.054
Perturb:experience	1.03	0.89	0.302
Perturb(control):learning:period	− 2.26	0.40	**0.024**
Perturb(perturbed): learning:period	14.70	0.28	**< 0.001**
Perturb(control):experience:period	− 9.44	0.09	**< 0.001**
Perturb(perturbed):experience:period	− 5.83	0.09	**< 0.001**
Random effects			
*σ*^2^	0.03		
*τ*_00_ _Individual:Pen_	1.29		
*τ*_00_ _Pen_	0.01		
ICC _Individual:Pen_	0.97		
ICC _Pen_	0.01		
Observations	221		
Marginal *R*^2^/conditional *R*^2^	0.310/0.985		

For factors, Perturb is set to Perturbed; Sex is set to Male; Period is set to the Second Period; unless specified in parentheses

Significant values (P < 0.05) are indicated in bold

**Fig. 4:**
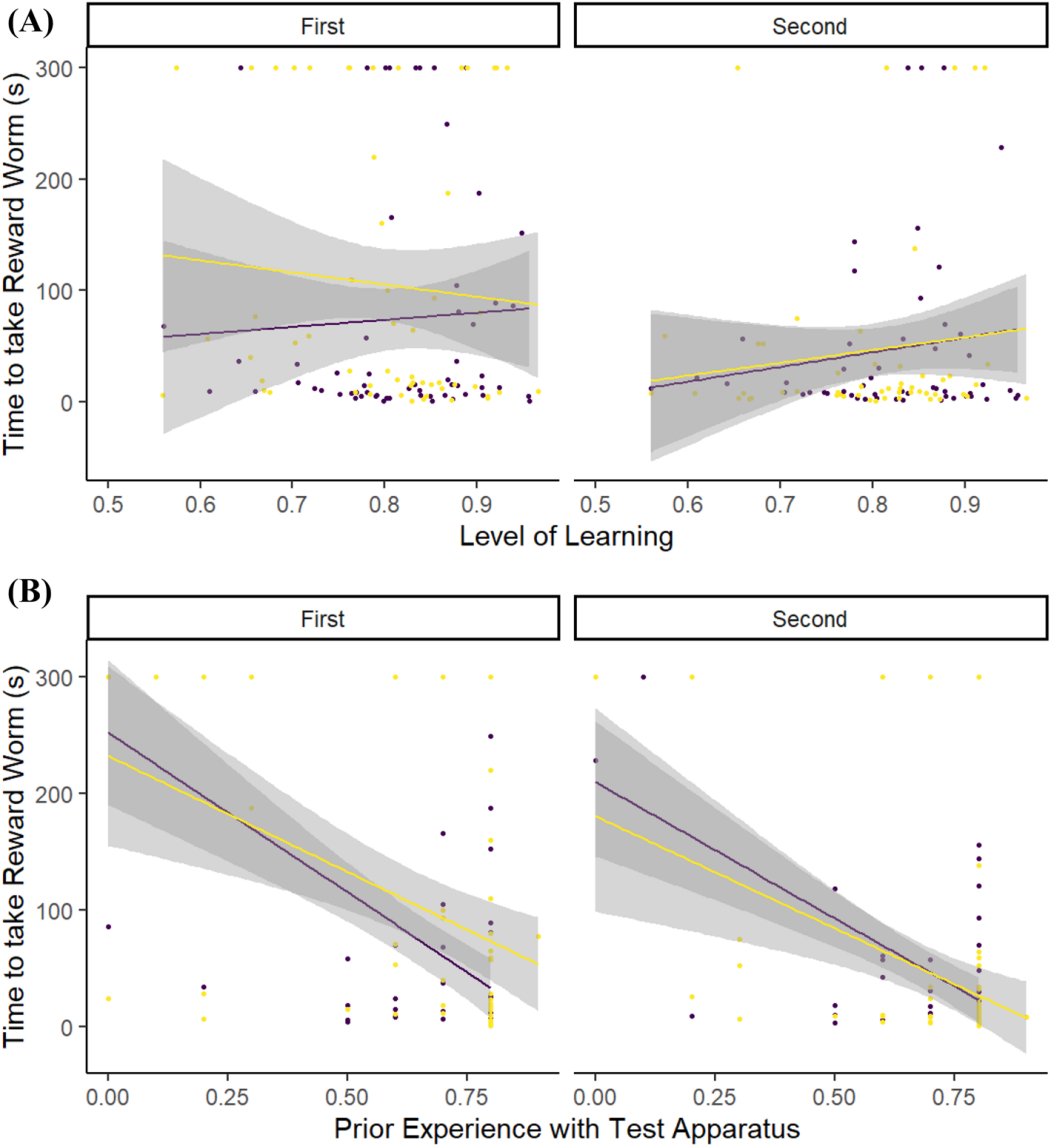
The relationship between the time that it took a focal individual to take and eat the reward worm from within the detour test apparatus during the two testing periods and: **a** the level of learning that the individual had achieved by the end of the preceding training period; **b** the level of experience that the individual had with the detour apparatus during the preceding training periods (value is the raw experience score/10). Individuals that had been perturbed immediately before the first test period are shown by Yellow (pale) lines and points. Control individuals that were not perturbed are shown by Purple (dark) lines and points. Lines indicate linear best fits. Shaded areas indicate 95% CI (color figure online)
4. What influenced the number of pecks an individual made at the apparatus?
  Individuals in the perturbed condition made more pecks at the detour apparatus than control individuals during the first testing period, with the strongest effect being seen in those individuals that had learned the discrimination less well. For control individuals, the effect was strongest for those that had learned the discrimination most accurately. This contrasted with their behaviour during the second testing period, when control individuals made more pecks, although this number decreased for those that had learned the discrimination most accurately (Table 3, Fig. 5a). Both perturbed and control birds made more pecks during the first testing period if they previously had more experience with the detour apparatus during the shaping sessions, but for control individuals, this relationship was reversed during the second testing period (Table 3, Fig. 5b). The decrease in numbers of unrewarded pecks between the first and second testing periods was significant for perturbed (post hoc Tukey test *p* < 0.0001), but not control individuals (post hoc Tukey test *p* = 0.158). Overall, thwarted individuals made more pecks at the detour apparatus than control individuals during the first period (post hoc Tukey test *p* < 0.0001), but both groups made similar numbers of pecks during the second period (post hoc Tukey test *p* = 0.065). Overall, males made more pecks at the detour apparatus than females, but body condition had no effect on number of pecks made (Table 3).

**Table 3:** Model output from a GLMM testing the relationship between the number of pecks that an individual made on the outside of a transparent cylinder containing a visible worm, and a suite of individual, temporal and environmental variables

Predictors	Peck		
	Estimate	SE	*p*
(Intercept)	1.83	0.87	0.067
Perturb	2.48	0.98	**0.013**
Learning	1.10	0.87	0.271
Experience	2.37	0.30	**0.018**
Sex	2.00	0.11	**0.045**
Condition	− 0.16	1.94	0.871
Period	4.87	0.44	**< 0.001**
Perturb:period	− 6.64	0.61	**< 0.001**
Perturb:learning	− 1.75	1.16	0.080
Perturb:experience	− 0.81	0.42	0.416
Perturb(control):learning:period	− 3.54	0.51	**< 0.001**
Perturb(perturbed): learning:period	2.38	0.48	**0.017**
Perturb(control):experience:period	− 4.85	0.25	**< 0.001**
Perturb(perturbed):experience:period	0.40	0.24	0.692
Random effects			
*σ*^2^	0.05		
*τ*_00_ _Individual:Pen_	0.24		
*τ*_00_ _Pen_	0.01		
ICC _Individual:Pen_	0.80		
ICC _Pen_	0.04		
Observations	221		
Marginal *R*^2^/conditional *R*^2^	0.329/0.888		

For factors, Perturb is set to Perturbed; Sex is set to Male; Period is set to the Second Period; unless specified in parentheses

Significant values (P < 0.05) are indicated in bold

**Fig. 5:**
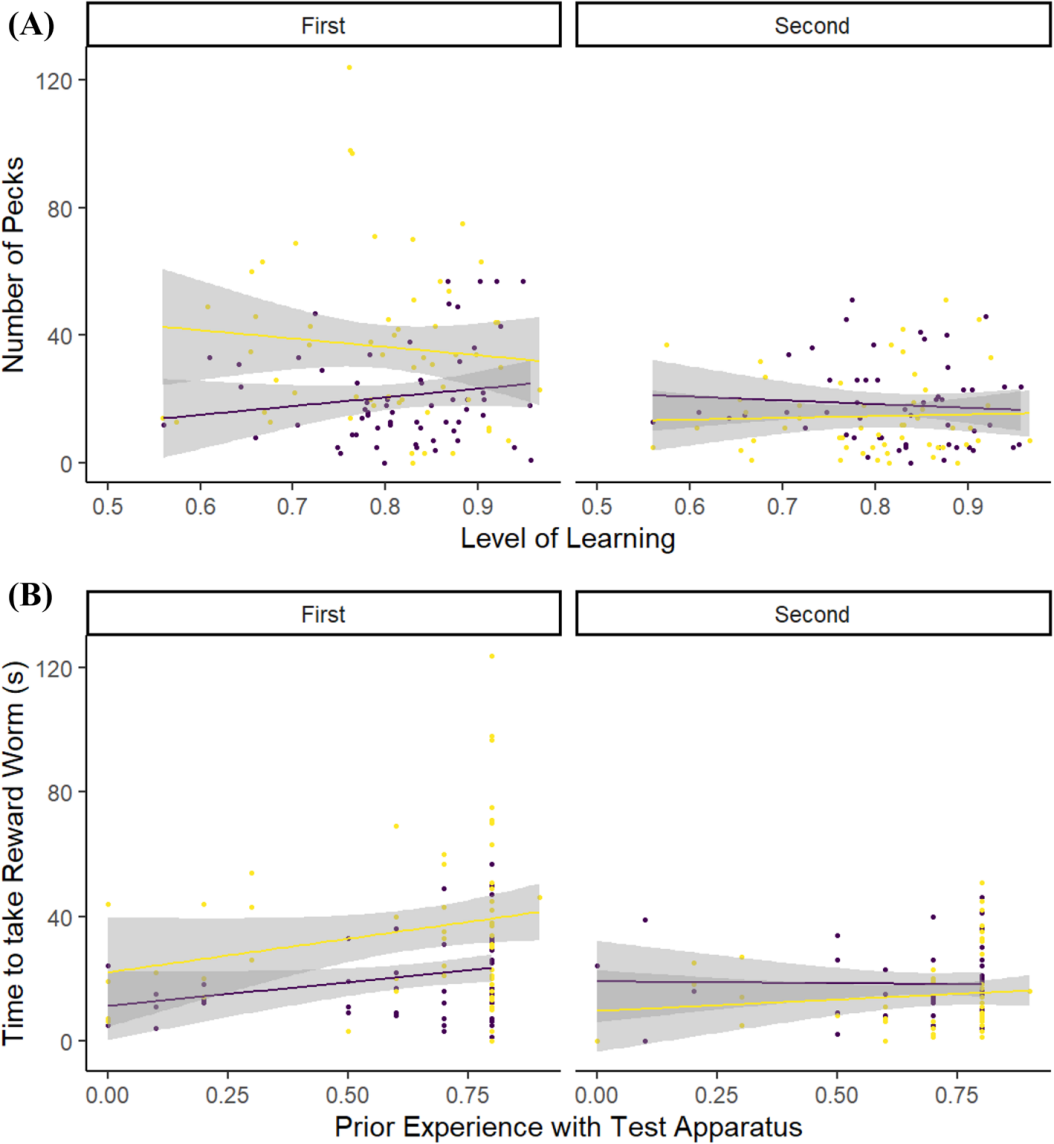
The relationship between the number of unrewarded pecks that a focal individual made to the detour apparatus during the two testing periods and: **a** the level of learning that the individual had achieved by the end of the preceding training period; **b** the level of experience that the individual had with the detour apparatus during the preceding training periods (value is the raw experience score/10). Individuals that had been perturbed during the first test period are shown by Yellow (pale) lines and points. Control individuals are shown by Purple (dark) lines and points. Lines indicate linear best fits. Shaded areas indicate 95% CI (color figure online)

## Discussion

An environment in which information was unpredictable, created by perturbing information in a learned binary discrimination task, altered pheasants’ performance on an inhibitory control task, causing them to increase the number of unrewarded pecks that they made at a transparent cylinder during a detour task presented immediately after they had experienced perturbed information, although they did not take longer to access the visible reward. Perturbed birds made ~ 30% more pecks to obtain the reward than those which did not have their information perturbed. This effect was immediate, occurring within seconds of the perturbation, and could not be explained by differences in satiation levels. However, the effect did not persist for long and a second presentation of the detour task immediately after a predictable discrimination session 3 days later revealed that, for perturbed birds, the number of unrewarded pecks made and the total time taken to access the reward fell to levels similar to those of control individuals. For perturbed birds, those that had learned the affordances of the discrimination task most accurately made fewer unrewarded pecks and accessed the reward worm more rapidly during the first test period. This negative relationship was not seen in control individuals. For both sets of individuals, those that had the greatest previous experience with the detour apparatus during the shaping blocks made more unrewarded pecks but accessed the reward worm more rapidly during the first test period. Non-cognitive factors, such as sex and body condition, also appeared to influence performance regardless of experimental treatment.

Pheasants that experienced a perturbation of an associative rule, specifically changed affordances of a binary colour discrimination task, made more unrewarded pecks at a transparent cylinder during the first test block. This change of task affordances mimics an environment in which rules about the features within it, specifically the accuracy of information, are unpredictable. Unpredictable environments can adversely influence cognitive abilities, including IC (Kotrschal and Taborsky 2010; Mittal et al. 2015). However, we found previously that making unpredictable the spatial environment in which a pheasant grew up led to increased IC in similar detour tasks compared to control birds (van Horik et al. 2019). This may have been because the habitat perturbation was actually enriching, which benefited the birds’ cognitive development, or it may be that different forms of unpredictability have different effects on IC performance. A consideration of just the two forms of unpredictability that we have induced is insufficient to differentiate these two hypotheses.

Why might a rapid perturbation of information in the environment immediately reduce an individual’s IC? One explanation for the increased pecking response of the perturbed group could be similar to the burst in response seen after an extinction phase in operant conditioning (Lattal and Lattal 2012; Lerman and Iwata 1995). By making the previously rewarded wells inaccessible, we began an extinction of the learned rule on the colour discrimination task. This could have induced birds to peck more or harder, not at the well apparatus but rather at the detour cylinder that was immediately presented. If the perturbation had initiated extinction of the learned rule then we might expect that birds in this condition might be less likely to make correct choices in subsequent tests. However, when we analysed the choices of all birds in their 10th block, the perturbed and control birds did not differ in their number of correct choices (*p* = 0.271), providing no evidence of extinction among the perturbed birds. A second explanation for increased pecking and/or decreased IC is that a perturbed environment may be strange and so generally arousing, perhaps because it can prove to be frustrating (Amsel 1958). Therefore, the effect of perturbation on IC may be mediated by an individual’s level of arousal, as predicted by the Yerkes–Dodson hypothesis (1908). Pet dogs that exhibited increased arousal performed worse in an IC detour task (unless they had been bred for calmness) (Bray et al. 2015). The effect of frustration adds a potential confound to our experiment that we did not initially anticipate. Even though we compensated perturbed birds for the loss of reward opportunity by providing freely accessible mealworms during the perturbation block, we could not disentangle the effects of frustration from that of information reliability. If information becomes unreliable, this may inevitably induce frustration, inducing decreases in IC rather than the individual adaptively adjusting their IC in response to altered informational circumstances. Our experiment cannot separate these two explanations. It is notable that perturbation only changed the number of unrewarded pecks that an individual made, rather than the time it took them to attain the reward (although perturbed birds did tend to take longer, but not significantly so), even though the number of pecks that an individual made and their time taken to attain the reward were correlated. Both measures have previously been used to infer IC (e.g. Lucon-Xiccato and Bisazza 2017; Vernouillet et al. 2018). If the effect of perturbation was to alter arousal, then it appears that this might have affected the level of arousal, indicated by amount of pecking exhibited, rather than the duration of arousal, indicated by the time which an individual would perseverate for.

The extent to which an individual had learned the rule which was subsequently perturbed affected their performance in the IC test. The individuals’ expectations were established by the extent to which they learned a colour discrimination rule. At a population level, 65% of birds had a probability of choosing the correct colour on their last binary choice above 0.80. However, as in previous similar binary choice tasks for pheasants (van Horik et al. 2018c), there was variation between individuals in the extent to which they had learnt the task as indicated by the range of probabilities of making a correct choice on the 80^th^ discrimination. In our study, inter-individual differences in learning were related to differences in the amount of unrewarded pecking and the speed of accessing the reward during the IC test, but only after their expectations had been perturbed. Among these birds, those that had learned the discrimination rule most accurately made fewer unrewarded pecks and accessed the reward worm more rapidly during the first test period. One might expect that these individuals had the most strongly established rules and hence that if these rules were perturbed, they might be most frustrated, manifesting in weaker IC. Our results contradict this prediction. Instead, the better learners were also those that exhibited the stronger IC. One explanation is that this could be explained by a single, or general, learning speed ability: those that better learned the discrimination were also more behaviourally flexible and so could more rapidly switch from an unrewarding pecking at the clear tube to an alternative method of moving and reaching into the tube. Relationships between associative learning and performance in detour tasks have been reported in other bird species but may take various directions and are seldom significant (Boogert et al. 2011; Guillette et al. 2015; Isden et al. 2013; Shaw et al. 2015). We suspect that a general learning ability is not the most likely explanation in our study because our previous work has found little support for general cognitive abilities either across or within particular domains in pheasants (van Horik et al. 2018b, c). Further, we did not find the same pattern among the control individuals, but rather those that had learned the task more accurately tended to take longer and make more pecks during the first test period.

The effects of perturbation were short-lived. As seen in other repeated tests of IC (Kabadayi et al. 2016, 2017; van Horik et al. 2018a) individuals improved across testing such that the time to obtain the reward in the second test period was ~ 52% less of that in the first and pheasants in the second period made ~ 58% of the pecks they did in the first period. Specifically, the times to obtain the reward and number of unrewarded pecks made by perturbed individuals fell to levels similar to those of control individuals during the second testing period. This suggests that an individual’s IC is plastic and highly susceptible to the immediate context in which it is being tested. Context specificity was also reported in domestic dogs (Bray et al. 2014). This highlights the importance of standardising the conditions immediately prior to testing an animal in order to measure its IC performance.

Studies of IC need to account for individuals’ prior experiences as these can influence their behaviour with the testing apparatus (Jelbert et al. 2016; van Horik et al. 2018a; Vernouillet et al. 2016). We found that the amount of prior experience an individual had during the shaping blocks (in terms of the number of blocks in which they obtained the reward worm from the opaque version of the detour apparatus) influenced their behaviour during the first testing block. Individuals with more experience with the opaque cylinder were faster to interact with the transparent cylinder during the first testing block, suggesting that they were less neophobic of the apparatus. Individuals with more experience were also faster to obtain the reward worm during the first testing block, after they had taken the baseline worm. This could again be because they were less neophobic and/or it could be because prior experience had given them a better understanding of the structure and affordances of the detour apparatus and hence they were faster to move to the open end to access the reward. However, individuals with more experience also made more unrewarded pecks during the testing block, suggesting that they might not necessarily have a better understanding of the apparatus but were either less neophobic and/or that they more strongly associated the apparatus with a reward and hence persisted in attempts to access the reward despite being blocked by the clear cylinder.

Several non-cognitive factors predicted an individual’s performance in the testing blocks. Individuals with a high body condition score took longer to take the baseline worm and interacted less with the opaque cylinder during the shaping period. If high body condition indicates low levels of hunger then perhaps hunger acts as a motivation to engage in the task. However, body condition did not explain differences in individuals’ performances in terms of the number of pecks they made or the time taken to access the reward worm during the testing block. Males made ~ 18% more pecks than females at the transparent cylinder. This contrasts with our previous findings where sexes did not differ in performance at barrier detour tasks (van Horik et al. 2018a) and although it matches findings in guppies (*Poecilia reticulata*), with females detouring round a transparent barrier faster than males (Lucon-Xiccato and Bisazza 2017), it does not match work on Clark’s nutcrackers (*Nucifraga columbiana*), which exhibited no sex differences (Vernouillet et al. 2016). We are not sure why we detected a sex difference in this task, but not in previous studies using the same apparatus.

The strength of inhibitory control that an individual exhibits appears to be susceptible to recent, short-term changes in information within the environment, but this effect is temporary and may be less marked in individuals that are generally more accurate learners. Although our work used pheasants as a model system, if these results are representative across taxa, then we suggest that some portion of individual differences observed during deployment of a detour or other IC task may be explained by an individual’s recent experiences of unexpected perturbation of their environment or the information available within it, mediated by their other cognitive abilities such as learning performance. This could cause problems for studies that aim to examine the causes and consequences of individual differences in IC if prior experience of local information reliability and other cognitive abilities are unknown. The results also suggest that when in an unpredictable environment or when information is changeable then individuals may benefit from refraining from, or delaying engaging in, tasks that require IC as they may perform poorly for a short period.

## Electronic supplementary material

Below is the link to the electronic supplementary material.

Supplementary material 1 (CSV 24 kb)

Supplementary material 2 (CSV 24 kb)

Supplementary material 3 (CSV 4 kb)

Supplementary material 4 (DOCX 129 kb)

Supplementary material 5 (R 12 kb)

## Data Availability

Data and r scripts are supplied as ESM.
