# Supplementary material for: The inhibitory control of pheasants (Phasianus colchicus) weakens when previously learned environmental information becomes unpredictable
Source: Anim Cogn. 2019 Dec 16;23(1):189–202. doi: 10.1007/s10071-019-01328-4 (PMC6981107; doi:10.1007/s10071-019-01328-4)
Supplement: Supplementary file 4 — Supplementary material 4 (DOCX 129 kb) [file 10071_2019_1328_MOESM4_ESM.docx]

**ESM for:**

**The inhibitory control of pheasants (*Phasianus colchicus*) weakens when environmental information becomes unpredictable**

Kandace R. Griffin, Christine E. Beardsworth, Philippa R. Laker, Jayden O. van Horik, Mark A. Whiteside and Joah R. Madden*

**Analysis of a subset of birds that we can be more confident had actually elearned the affordances of the discrimination task.**

Our definition of learning is debatable. We considered all birds that had a >50% chance of making a correct choice on their final trial as exhibiting at least some learning of the task affordances. It may be more informative to consider only those birds that have exceeded a criteria considered to indicate that they have definitively learnt the task. We also adopted this more conservative approach and repeated all of our analyses on a subset of 59 birds that exhibited a probability of >80% of making the correct choice on their 80^th^ discrimination. These analyses correspond to Results 2), 3) & 4), with the associated model output Tables and Figures,

A)


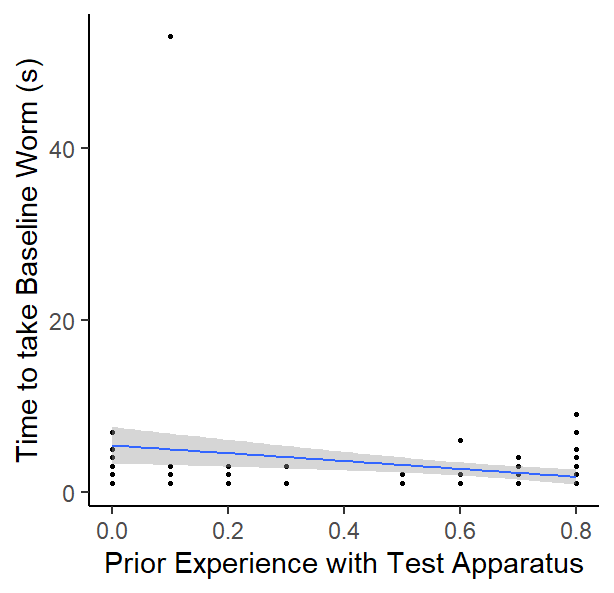


B)


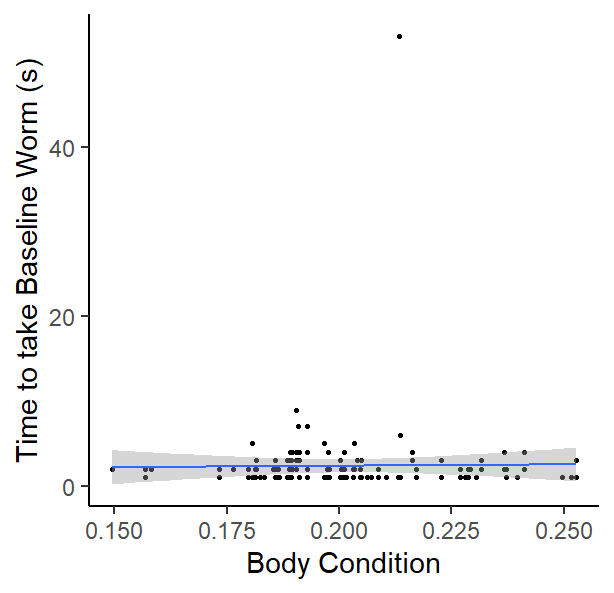


**Figure 1** The relationship between the time that it took a focal individual that had a >80% probability of making a correct choice on their final choice to take and eat the baseline worm placed on the detour test apparatus during their testing periods and: A) the level of experience that the individual had with the detour apparatus during the preceding shaping blocks (value is the raw experience score/10); B) their body condition (value is the raw body condition score x 100). Lines indicate linear best fits. Shaded areas indicate 95% CI.

|  | **BW** | | |
| --- | --- | --- | --- |
| *Predictors* | *Incidence Rate Ratios* | *std. Error* | *p* |
| (Intercept) | 29.84 | 2.86 | 0.235 |
| Thwarted | 0.00 | 4.48 | 0.160 |
| X.80 | 0.20 | 3.06 | 0.601 |
| ScaleTotInt | 0.09 | 0.44 | **<0.001** |
| M | 1.15 | 0.18 | 0.442 |
| ScaleCond | 3.18 | 4.17 | 0.781 |
| Second | 0.02 | 3.14 | 0.202 |
| ThwartedThwarted:TestSecond | 205.74 | 5.15 | 0.301 |
| ThwartedThwarted:X.80 | 294.78 | 5.05 | 0.260 |
| ThwartedThwarted:ScaleTotInt | 4.10 | 0.74 | 0.056 |
| ThwartedControl:X.80:TestSecond | 8.69 | 3.66 | 0.555 |
| ThwartedThwarted:X.80:TestSecond | 0.33 | 4.51 | 0.808 |
| ThwartedControl:ScaleTotInt:TestSecond | 26.28 | 0.56 | **<0.001** |
| ThwartedThwarted:ScaleTotInt:TestSecond | 1.20 | 0.64 | 0.771 |
| **Random Effects** | | | |
| σ^2^ | 0.42 | | |
| τ_00_ _Bird:Pen_ | 0.19 | | |
| τ_00_ _Pen_ | 0.00 | | |
| N _Bird_ | 69 | | |
| N _Pen_ | 4 | | |
| Observations | 137 | | |
| Marginal R^2^ / Conditional R^2^ | 0.314 / NA | | |

**Table 1** Model output from a GLMM testing the relationship between the time to take a baseline worm and a suite of individual, temporal and environmental variables for individuals that had a >80% probability of making a correct choice on their final choice. For factors, Perturb is set to Perturbed; Sex is set to Male; Period is set to the Second Period; unless specified in parentheses

A)


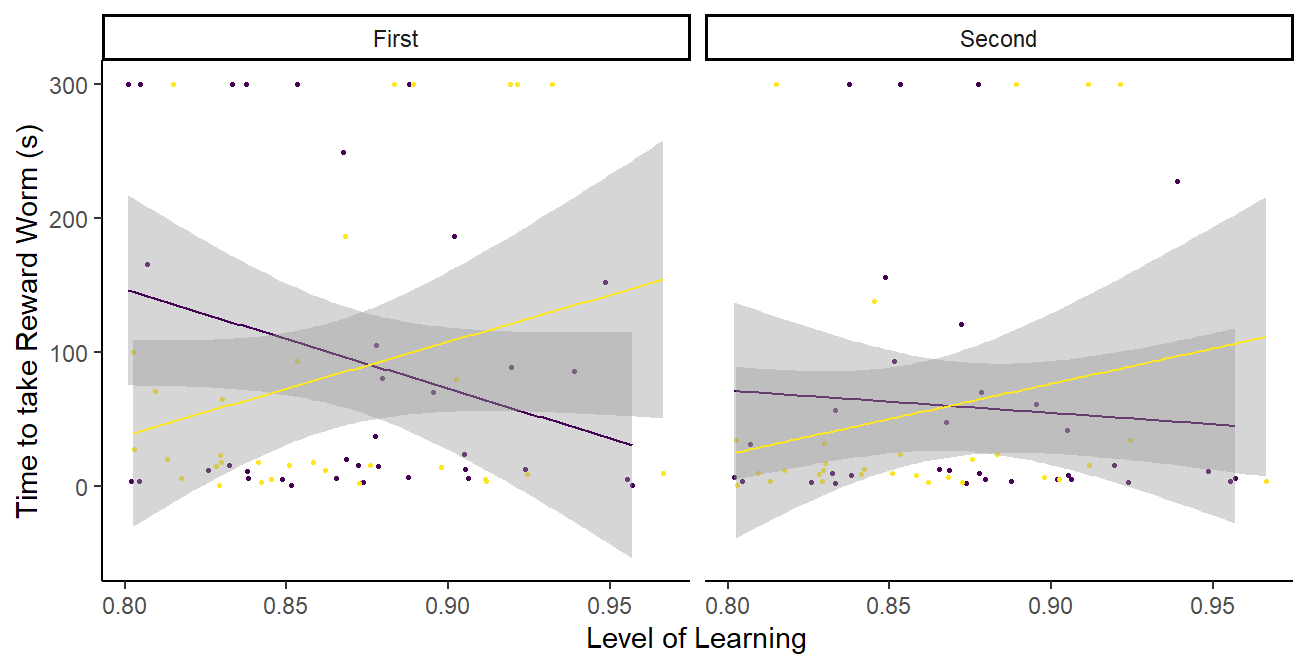


B)


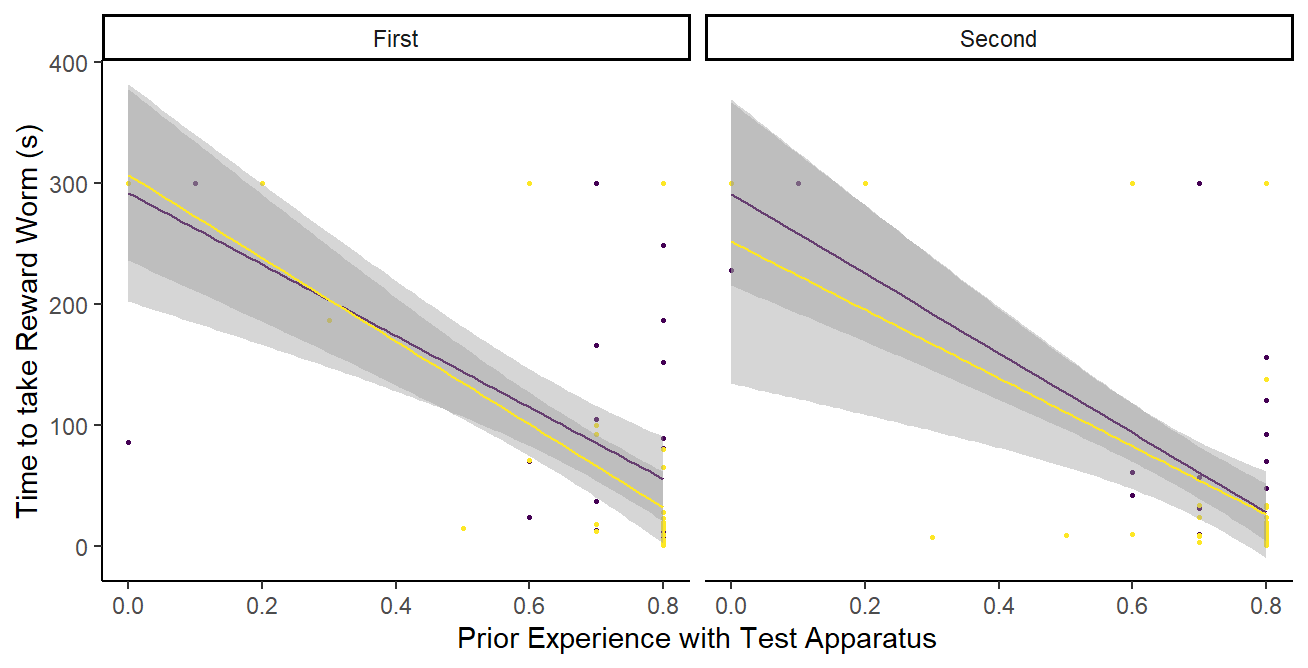


**Figure 2** The relationship between the time that it took a focal individual that had a >80% probability of making a correct choice on their final choice to take and eat the reward worm from within the detour test apparatus during the two testing periods and: A) the level of learning that the individual had achieved by the end of the preceding training period; B) the level of experience that the individual had with the detour apparatus during the preceding training periods (value is the raw experience score/10). Individuals that had been perturbed immediately before the first test period are shown by Yellow lines and points. Control individuals that were not perturbed are shown by Purple and points. Lines indicate linear best fits. Shaded areas indicate 95% CI.

|  | **Latency** | | |
| --- | --- | --- | --- |
| *Predictors* | *Incidence Rate Ratios* | *std. Error* | *p* |
| (Intercept) | 357550.69 | 4.12 | **0.002** |
| Thwarted | 0.00 | 5.62 | 0.332 |
| X.80 | 0.00 | 4.33 | 0.159 |
| ScaleTotInt | 0.04 | 0.75 | **<0.001** |
| M | 0.86 | 0.29 | 0.612 |
| ScaleCond | 0.00 | 6.64 | 0.268 |
| Second | 0.03 | 0.73 | **<0.001** |
| ThwartedThwarted:TestSecond | 0.14 | 1.02 | 0.054 |
| ThwartedThwarted:X.80 | 436.39 | 6.38 | 0.340 |
| ThwartedThwarted:ScaleTotInt | 0.92 | 1.09 | 0.939 |
| ThwartedControl:X.80:TestSecond | 82.03 | 0.84 | **<0.001** |
| ThwartedThwarted:X.80:TestSecond | 445.53 | 0.83 | **<0.001** |
| ThwartedControl:ScaleTotInt:TestSecond | 0.31 | 0.09 | **<0.001** |
| ThwartedThwarted:ScaleTotInt:TestSecond | 0.90 | 0.11 | 0.323 |
| **Random Effects** | | | |
| σ^2^ | 0.03 | | |
| τ_00_ _Bird:Pen_ | 1.17 | | |
| τ_00_ _Pen_ | 0.00 | | |
| N _Bird_ | 69 | | |
| N _Pen_ | 4 | | |
| Observations | 132 | | |
| Marginal R^2^ / Conditional R^2^ | 0.966 / NA | | |

**Table 2** Model output from a GLMM testing the relationship between the time taken (s) to access the reward worm placed inside a transparent cylinder, and a suite of individual, temporal and environmental variables for individuals that had a >80% probability of making a correct choice on their final choice. For factors, Perturb is set to Perturbed; Sex is set to Male; Period is set to the Second Period; unless specified in parentheses.

A)


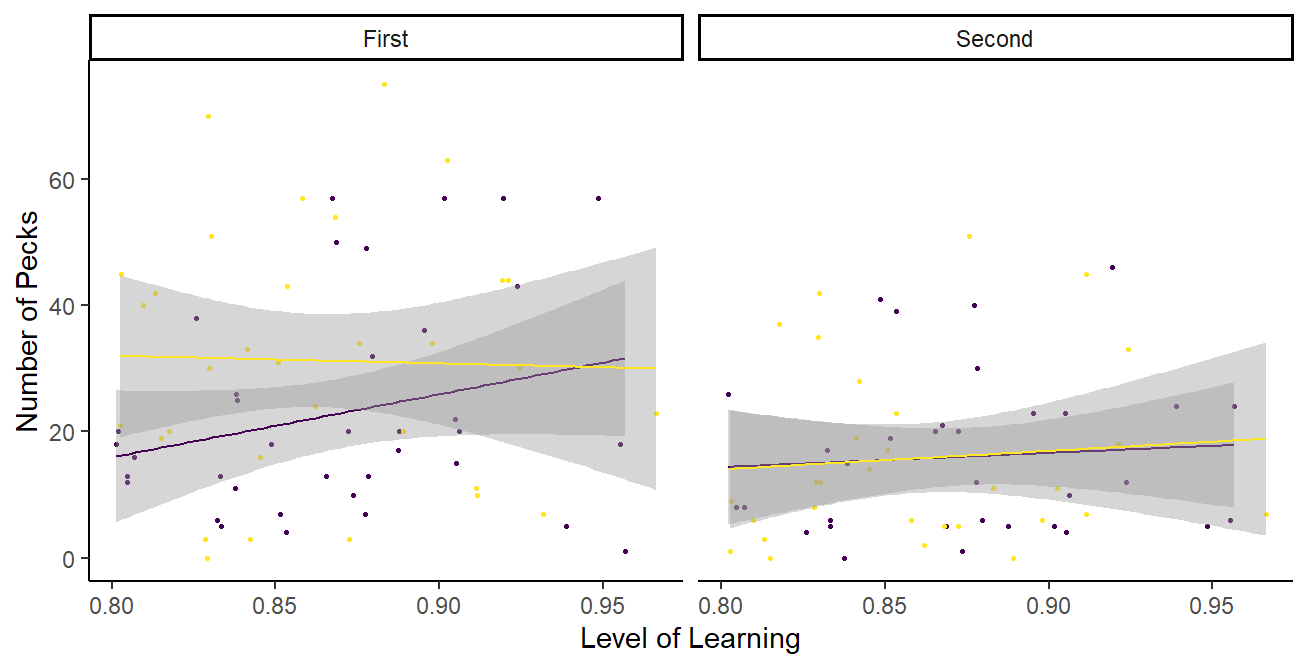


B)


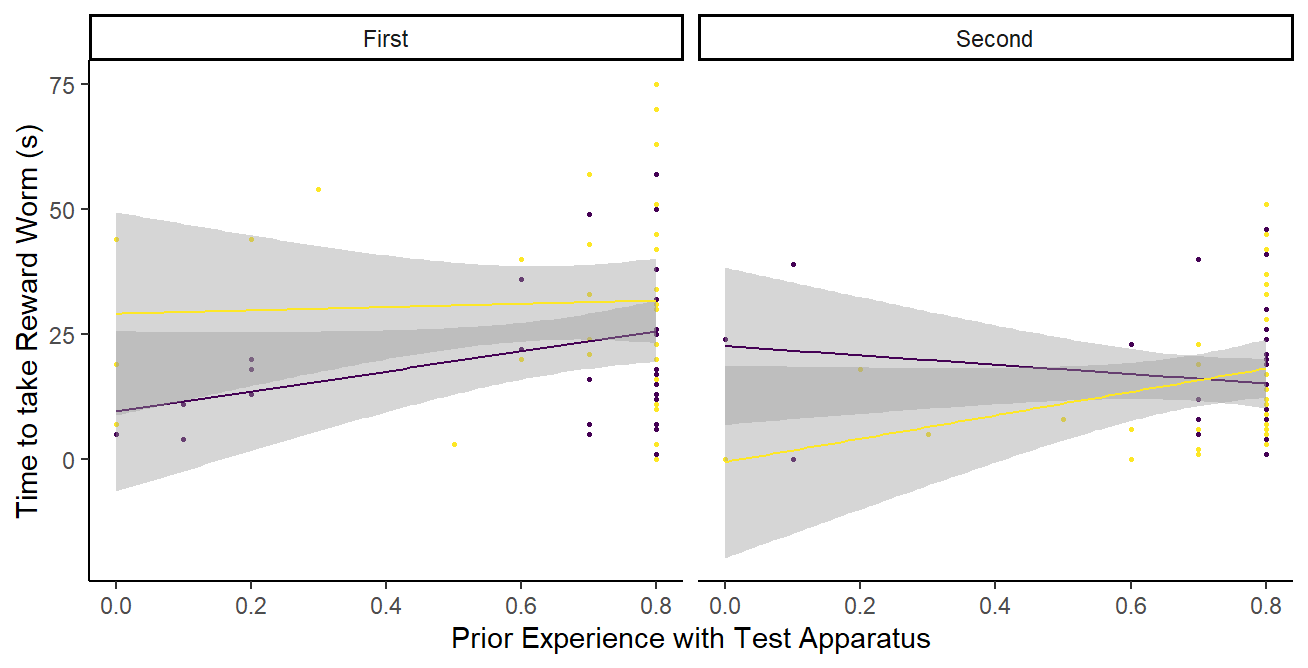


**Figure 3** The relationship between the number of unrewarded pecks that a focal individual with a >80% probability of making a correct choice on their final choice made to the detour apparatus during the two testing periods and: A) the level of learning that the individual had achieved by the end of the preceding training period; B) the level of experience that the individual had with the detour apparatus during the preceding training periods (value is the raw experience score/10). Individuals that had been perturbed during the first test period are shown by Yellow lines and points. Control individuals are shown by Purple lines and points. Lines indicate linear best fits. Shaded areas indicate 95% CI.

|  | **Peck** | | |
| --- | --- | --- | --- |
| *Predictors* | *Incidence Rate Ratios* | *std. Error* | *p* |
| (Intercept) | 0.36 | 2.09 | 0.624 |
| Thwarted | 226.53 | 2.89 | 0.060 |
| X.80 | 68.82 | 2.21 | 0.056 |
| ScaleTotInt | 2.80 | 0.41 | **0.012** |
| M | 1.29 | 0.15 | 0.092 |
| ScaleCond | 0.08 | 3.26 | 0.427 |
| Second | 81.01 | 1.30 | **0.001** |
| ThwartedThwarted:TestSecond | 0.00 | 1.83 | **<0.001** |
| ThwartedThwarted:X.80 | 0.01 | 3.27 | 0.120 |
| ThwartedThwarted:ScaleTotInt | 0.39 | 0.57 | 0.100 |
| ThwartedControl:X.80:TestSecond | 0.02 | 1.44 | **0.008** |
| ThwartedThwarted:X.80:TestSecond | 12.81 | 1.44 | 0.076 |
| ThwartedControl:ScaleTotInt:TestSecond | 0.13 | 0.31 | **<0.001** |
| ThwartedThwarted:ScaleTotInt:TestSecond | 10.85 | 0.42 | **<0.001** |
| **Random Effects** | | | |
| σ^2^ | 0.05 | | |
| τ_00_ _Bird:Pen_ | 0.26 | | |
| τ_00_ _Pen_ | 0.02 | | |
| ICC | 0.84 | | |
| N _Bird_ | 69 | | |
| N _Pen_ | 4 | | |
| Observations | 132 | | |
| Marginal R^2^ / Conditional R^2^ | 0.384 / 0.901 | | |

**Table 3** Model output from a GLMM testing the relationship between the number of pecks that an individual with a >80% probability of making a correct choice on their final choice made on the outside of a transparent cylinder containing a visible worm, and a suite of individual, temporal and environmental variables. For factors, Perturb is set to Perturbed; Sex is set to Male; Period is set to the Second Period; unless specified in parentheses.
